# An Inspiring Address

**Published:** 1916-10-28

**Authors:** 


### The Hospital

The Workers' Newspaper of Administrative Medicine and Institutional Life, Administration, National Insurance and Health.

No. 1586, Vol. LXI. SATURDAY, OCTOBER 28, 1916. PRICE ONE PENNY.

### AN INSPIRING ADDRESS.

The Presidential Address by Dr. Winford H. Smith which we publish in our present issue is a document of much interest and importance. Though directly addressed to an audience on the other side of the Atlantic it touches many questions which are urgent in this country. Further, it is spoken by an authority who knows his subject from a personal experience gained not only in the administration of one of the most famous of hospitals, but also by a broad study of the whole hospital question. And yet again, the address is characterised by many engaging qualities. It deals with great principles and not with mere details. It is characterised by thoroughness and lucidity. Above all, it is frank, outspoken, and sincere. Instead of the commonplace compliments and smooth sayings which so often characterise ceremonial oratory we have here candid criticism and the exposure of weaknesses and failures, attended, however, with helpful suggestions for improvement. If some ears are made to tingle at the sound of its censures, we may all with advantage remember that "faithful are the wounds of a friend."

Our own sympathies are particularly attracted by the demand which Dr. Smith makes for a recognition of the important civic position which the hospital ought to occupy. Its primary function is obvious and is universally accepted. Moreover, no activities may be undertaken which hinder or come into conflict with this function. But the efficient medical and surgical care of the sick and suffering poor, though standing as the first duty of a hospital, is not inconsistent with other ambitions. Indeed, when the subject is rightly viewed other ambitions naturally and properly grow out of this duty. For, the discharge of the duty gives opportunities, and opportunities in their turn bring responsibilities and obligations. Too often in the minds of many members of the community the hospital excites a mere sentimental tenderness as a place in which unfortunate citizens are sheltered during periods of suffering and ill-health. Medical treatment is afforded and a gentle benevolence is practised. Hence, on account of its philanthropic and charitable enterprises the hospital is classed as an institution engaged in "good works" and in an endeavour to relieve the suffering which for some mysterious but doubtless wise purpose has fallen upon our poorer neighbours. Dr. Smith's address is a stimulating challenge to this narrow and restricted view. He presents the hospital as a large factor and one charged with many functions in the life of the community, and he claims for it the dignity and wide interest which are the necessary outcome of this position. It is with a ready response that we listen to his argument that the hospital ought to take its place in an organised Health Service, and ought to be linked with other organisations to secure this end. Even for its immediate purpose—the re-establishment of the health and personal value of its patients—the present isolation and detachment of the hospital curtail and leave incomplete the fulness of its work. The value of the country convalescent home in this relation is generally accepted, though in not a few instances it remains a dream rather than a reality. But something more is necessary if the work of the hospital is to bear its full fruit. Though disease may have been checked by appropriate treatment in the wards there are not a few cases in which if the patient has to return to his former environment he will ere long again be a sufferer. What he needs if he is to have anything like a chance of health and activity is a new condition of life. The loss here is not merely to the individual. Equally it is a loss in the sphere of national economics. For practical purposes the work of the hospital is largely lost and wasted because no efficient protection exists for the discharged patient, cured in a sense, but unfit for the burden of his former occupation. The hospital cannot supply this protection, and it is not within the sphere of its duty; but it can make the want known.

Still more broadly the whole of the health question is in the hospital sphere, and no organisation is more favourably situated for its public presentation. Official schemes concerned with preventive medicine are in their working more or less formal and even bureaucratic, and they hardly touch the popular sympathies. But the hospital has the opportunity of exercising a large educative influence in reference to the whole subject of health, because its management is less official than popular and because its voluntary principle has none of the forbidding characters of compulsory methods. The general body of the people when educated to the opportunity have the disposition to co-operate, whilst a cold assent is all that is to be expected in the case of legal enactments. With unqualified cordiality we support Dr. Smith's invitation to our hospital authorities to undertake a mission which will put before the community the true position which the hospital ought to hold in the civic and national life.

All this necessarily involves the supposition that the hospitals themselves in all their departments are ready to meet the full measure of the claims which fall upon them. As their first function is curative they must be ready for this with the most efficient officers and the most serviceable equipments. But, in addition, all concerned in their working must be alive to the chances of medical and surgical advance, and the staff must justify their opportunities by the use they make of them. Further, there must be a determined effort to get beyond a mere local and isolated benevolence in order to attain a position which will make the special knowledge gained in the hospital a part of the machinery by which the health of the community is protected and secured.

